# Taurine and proliferation of lymphocytes in physically restrained rats

**DOI:** 10.1186/1423-0127-17-S1-S24

**Published:** 2010-08-24

**Authors:** Fili Fazzino, Francisco Obregón, Lucimey Lima

**Affiliations:** 1 Laboratorio de Neuroquímica, Centro de Biofísica y Bioquímica, Instituto Venezolano de Investigaciones Científicas-Altos de Pipe-Km 11, Carretera Panamericana, Caracas, Miranda, Venezuela

## Abstract

**Background:**

Taurine is present in lymphocytes and seems to modulate certain immune cell functions. Among the effects of taurine on these cells are protection against antioxidants and regulation of inflammatory aspects of the immune response. Stress affects antigen presentation, traffic and proliferation of leukocytes, as well as antibody and cytokine secretion. The purposes of this study were to explore the possible direct effects of taurine concentrations on lymphoproliferation and interleukins levels in control and in physical restrained rats.

**Methods:**

Lymphocytes of male Sprague-Dawley rats, stressed by physical restrain and controls (5 h per day for 5 days) were isolated from blood by Histopaque (1077 g/l) and differential adhesion to plastic, and then cultured (72 h) in the presence of different concentrations of taurine (0.5 – 50 mM), β-alanine (0.5 – 50 mM), or both, without or with the T cells mitogen, concanavalin A. Plasma and lymphocytes levels of pro-inflammatory interleukin-1β and anti-inflammatory interleukin-10 were respectively measured by Pierce Endogen rat ELISA Kits. Taurine in plasma and in lymphocytes were determined by HPLC.

**Results:**

Lymphoproliferation of resting cells significantly decreased in the presence of 3 and 6 mM taurine and increased up to control level at 12 mM taurine. In concanavalin A-activated lymphocytes, the effect of taurine was greater. β-alanine increased lymphoproliferation in a bell shaped dose-dependent manner and decreased it in activated lymphocytes but in a lower magnitude. In combination, β-alanine impaired the effect of taurine at 3 and 6 mM. After restriction, no change in lymphoproliferation was observed at different concentrations of the amino acids without or with concanavalin A, although pro-inflammatory interleukin and taurine in plasma and in lymphocytes significantly increased.

**Conclusions:**

Taurine affects lymphoproliferation in control rats, following a dose-dependent manner, an effect that might involve its transport into the cells. Elevation of interleukin-1β produced in stressed rats by physical restrain could seriously affect the immune balance, whereas taurine increase might be protective. These results suggest that taurine and taurine transport play a role in lymphoproliferation. In addition, modifications of taurine system in lymphocytes take place during restriction stress.

## Introduction

Taurine (TAU) and taurine transporter (TAUT) are present in lymphocytes [1,2]. One of the functions of TAU in these cells might be related to protection against oxidants [3], regulation of pro-inflammatory cytokines in humans and formation of taurine cloramine (Tau-Cl) [4]. Exogenous TAU was shown to possess significant anti-inflammatory properties in various *in vivo* and *in vitro* models of inflammation, and to protect the tissues by increasing the defending capacity of organs against oxidative damage in inflammatory bowel disease, pancreatitis, and gastric mucosal injury [5,6]. This anti-inflammatory action of TAU was shown to be a direct result of its antioxidant effects, which inhibits lipid peroxidation and neutrophil activation [5]. The effect of TAU in combating oxidative damage is well known to result from its ability to scavenge hypochlorous acid generated in neutrophils in the process of phagocytosis to form the relatively harmless Tau-Cl [7]. Tau-Cl inhibits the activation of nuclear factor κB, a potent signal transducer for inflammatory cytokines [8].

Physical restraint a well know stress model, increases oxidative processes [9], reduces T lymphocyte proliferation in response to concanavalin A (Con A), decreases the number of CD4^+^ T cell subpopulation without changes in CD8^+^ T cells, impairs T helper component of immunity [10], and could induce an elevation of plasma interkeukin 6, tumor necrosis factor-α (TNF-α) and interferon γ [11-13].

The purposes of this study were to explore the possible direct effects of TAU on lymphoproliferation as well as levels of TAU, and pro- and anti-inflammatory interleukins in physically restrained rats and its controls.

## Methods

### Animals and stress procedures

Male Sprague–Dawley rats (*Rattus norvegicus*) ranging in weight from 200 to 250 g were obtained from the hatchery of Instituto Venezolano de Investigaciones Científicas (IVIC). The animals were housed individually in a room controlled for temperature, humidity and lighting. Commercial rat food and water were available *ad libitum*. All manipulations followed international ethical guide [14]. Rats were stressed by restraint in an immobilization conical tube of 50 ml (restrainer) with ventilation holes for 5 hours and for 5 consecutive days. All stress procedures occurred from 11:00 am to 4:00 pm.

### Preparation of blood peripheral lymphocytes

The rats were anesthetized with ether and blood samples were taken by intracardiac puncture between 10:00 and 11:00 am in tubes with EDTA, 1.8 mg/ml. The blood was centrifuged at 1000 rpm with a vasculant rotor for 10 min at room temperature. The plasma was collected for determination of interleukins and amino acid analysis, and the layer of white cells plus some red blood cells was taken and transferred to tubes with 10 ml of isotonic saline 0.1 M sodium phosphate buffer pH 7.4 (PBS). These suspensions were placed on 3 ml of Histopaque (Sigma) (1077 g/l). After centrifugation at 2000 rpm for 30 min peripheral mononuclear cell layer was taken, washed twice with PBS and centrifuged at 1200 rpm for 10 min. To achieve enriched lymphocyte preparation with a minimal monocyte contamination. The resulting pellet was diluted with Roswell Park Memorial Institute Medium 1640 (RPMI) free of bovine serum albumin and incubated in a plastic flask for 45 min at 37° C and 5% of CO_2_. After the incubation, lymphocytes, which are non-adherent cells (80–90%), were dislodged from adherent monocytes, transferred to plastic tubes and washed twice. The integrity of isolated lymphocytes was determined by Trypan blue exclusion test and was greater than 90%.

### Lymphoproliferation assay

Lymphocytes were cultured in 96 well plaques, in which 200,000 cells were placed in each one to a final volume of 200 µl of RPMI medium with gentamicin (100 µg/ml), L-glutamine (2 mM) and 10% fetal calf serum (Gibco BRL, Maryland). The incubation was performed at 37° C, 5% CO_2_ and 100% humidity for 72 h in the absence or in the presence of Con A at suboptimal concentration, 2 µg/ml. TAU (1.5–24 mM) and β–alanine (β-Ala) (0.8-50 mM) ware added to the cultures. Proliferation was measured with 3-[4,5-dimetilazol-2-il]-2,5-diphenil-tetrazolio (MTT) (Sigma, St Louis, MO) (19,20). MTT was prepared in PBS, 5 mg/ml, 20 µl was added to each well, and incubation was done for 4 h at 37°C. Then, 100 µl of solution was extracted and 100 µl of HCl 0.04 N in isopropanol was added. After mixing, the plaque was read in a GENios lector (Tecan) at 570 nm with the Program Magellan.

### Measurement of plasma interleukin-1β and interleukin-10

Plasma collected for interleukin-1β (IL-1β) and interleukin-10 (IL-10) assays was stored at -80°C. The levels of IL-1β and IL-10 were measured by ELISA Endogen kits (Pierce Endogen, Cambridge, MA) following the manufacturer’s instructions. Briefly, 100 µl of samples were dispensed into 96 wells coated with rat IL-1β or IL-10 antibody and incubated for 2 hours at room temperature. After extensive washing, 100 µl of the biotinylated anti-IL-1β (or IL-10) were added to each well, and plates were incubated for 30 min at room temperature. The wells were again washed 5 times, 100 µl of Streptavidin-HRP was added and incubation was done for 30 min. 3,3´,5,5´-tetramethylbenzidine (TMB) (100 μL/well) was used as the chromogen for the colorimetric assay. The reaction was stopped by adding 100 µl/well of stop solution and the absorbance was read at 450 nm. The levels of ILs are expressed as pg/ml.

### Determination of taurine

TAU in plasma and in lymphocytes was determined by high performance liquid chromatography (HPLC) with fluorescent detection employing a modified method (16). The HPLC system consisted of a Waters 2690 Separation System and a Shimadzu RF-551 fluorescent detector. A Sulpeco LC-18 column 4.6 X 100 mm, 5 µm was employed for amino acid separation. Platelet poor plasma, 300 µl, was acidified with 50 µl of 20% sulfosalicylic acid. Centrifugation was carried out at 17,000 rpm for 20 min, at 4°C, and supernatant was kept at 80°C until chromatographic analysis. Immediately before injection, 50 µl of the supernatants plus 150 µl of potassium borate buffer pH 10.4 and 200 µl of the mixture: 25 mg o-phtaldehyde, 500 µl methanol, 25 µl β-mercaptoethanol (1 g/ml), and 4.5 ml 0.4 M potassium borate buffer pH 10.4 was used for derivatization. Then, 15 µl of the derivatized preparation were injected into the chromatographic system. The levels of amino acids were calculated from the area under the curve of samples and external standards with program Millenium, and expressed as nmol/ml.

### Analysis of data

Data are expressed as the arithmetic mean ± standard error of the mean (SEM). Differences were statistically analyzed using the Student's t-test. Statistical significance was considered if P < 0.05. Data management and statistical analysis were conducted employing the program Microsoft Office Excel 2007.

## Results and discussion

### Lymphoproliferation

**Figure 1:**
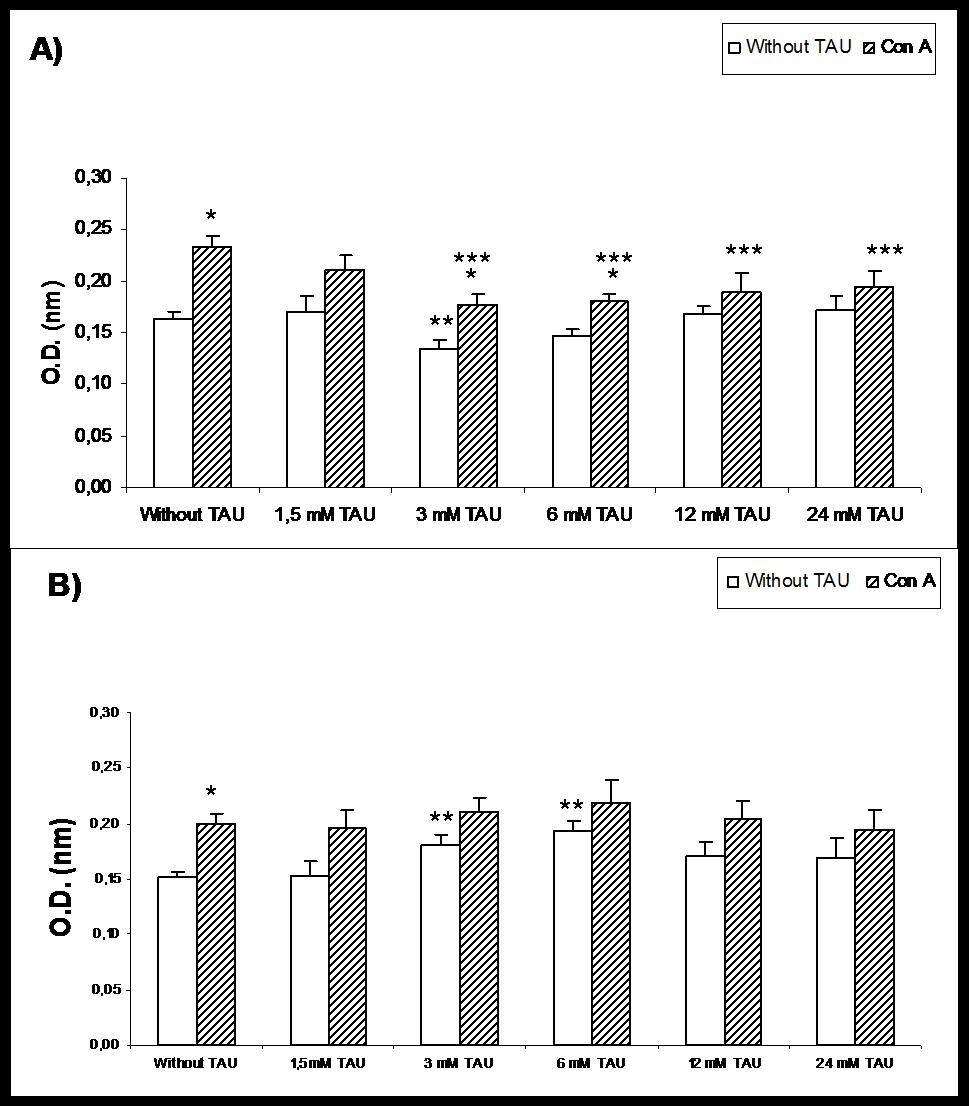
**Effect of taurine (TAU) on lymphoproliferation in the absence and in the presence of concanavalin A (Con A), A) in control rats, and B) in physical restrained rats.** Each value represents the mean ± SEM, *n* = 6. * P < 0.05 respecting corresponding Basal. ** P < 0.05 respecting Without TAU Basal. *** P < 0.05 respecting Without TAU Con A.

**Figure 2:**
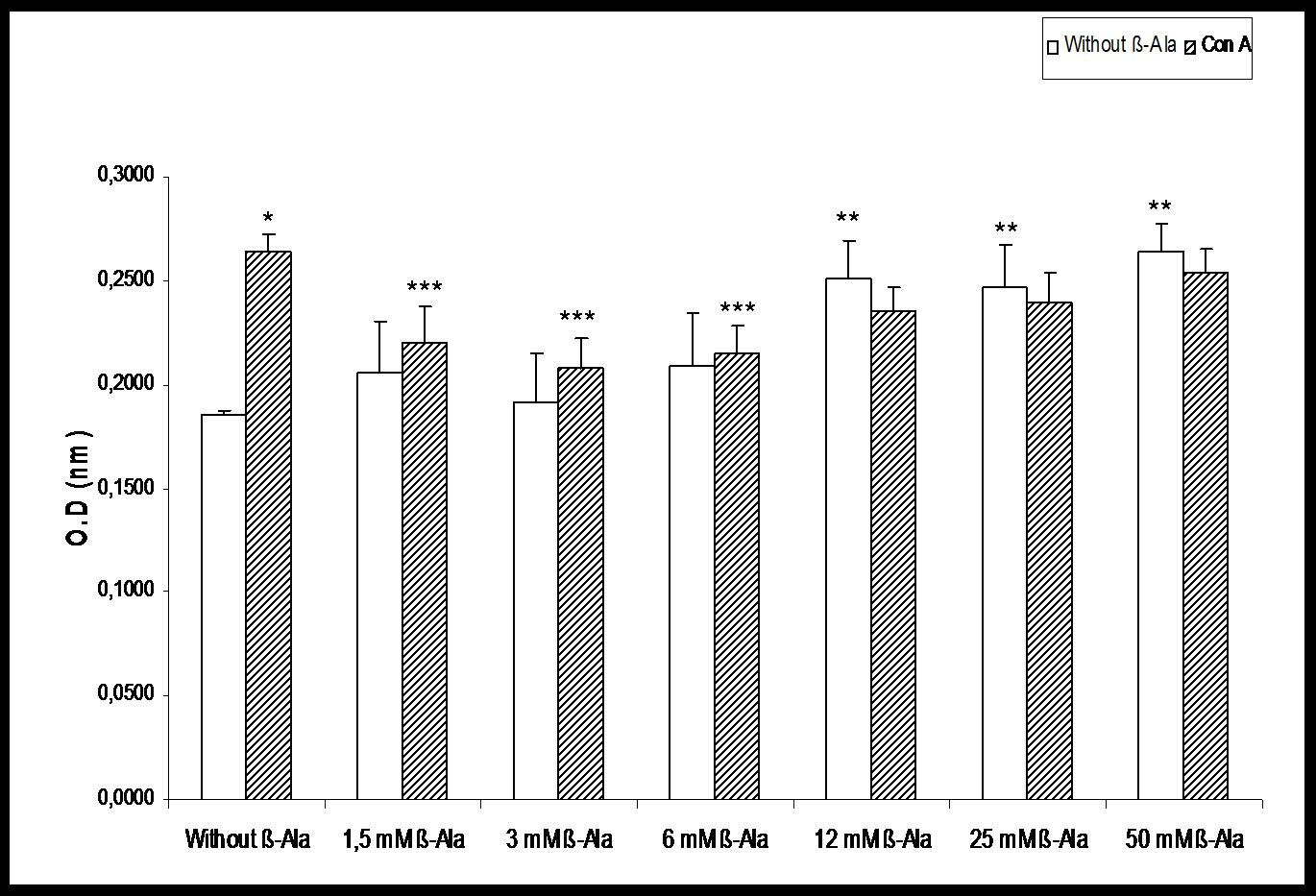
**Effect of β-alanine (β-Ala) on lymphoproliferation in the absence and in the presence of concanavalin A (Con A) in control rats.** Each value represents the mean ± SEM, *n* = 6. * P < 0.05 respecting corresponding Basal. ** P < 0.05 respecting Without β-Ala Basal. *** P < 0.05 respecting Without β-Ala Con A.

Figure 1A shows that lymphoproliferation of control rats significantly decreased in the presence of 3 mM TAU and reached basal levels at 6 mM TAU. While in Con A- activated lymphocytes, there is also a significant decrease a 3 mM, but it remains low in the following concentrations. In restrained rats (Figure 1B) there were no statistical changes in activated lymphocytes, and resting showed a significant increase at 3 and 6 mM, but returned to basal values at greater concentrations. The Figure 2 shows that to values above 6 mM β-Ala the lymphoproliferation significantly increased In Con A- activated lymphocytes, there was a significant decrease between 1,5 and 6 mM and reached basal levels unactivated. However, at higher concentrations when the values of lymphoproliferation were again statistically similar to basal of Con A, we observed that the effect decreased to a lesser extent in Con A-activated lymphocytes (Figure 2).

Protein kinase C (PKC) isoforms plays an important role in lymphoproliferation. PKC-θ, is a crucial regulator of T-cell activation and proliferation as well as of cytokine production [15-17]. Moreover, PKC-α is also involved in T-cell proliferation acting upstream of PKC-θ [18,19]. In addition, Tau has been shown to affect PKC isoforms, regulating their activity an expression in a concentration-dependent manner [20,21], and it is possible that the observed response in lymphoproliferation is due to the effect of Tau on PKC isoforms and/or inhibition of the nuclear translocation of NF-kappaB by inhibition of PKC-α expression [19]. However, TAU also regulates the phosphorylation of TAUT, through PKC as an adaptive response to changes in TAU availability [22-24], so maybe TAUT plays an important role in the lymphoproliferation.

On the other hand, since previous studies show that inhibitors of PKC suppress mitogen induced T-cell proliferation [25], it is possible that Tau acts as an inhibitor of PKC at concentrations above 3 mM, and therefore the effect in lymphoproliferation is more prominent in Con A activated- lymphocytes.

Previous studies have demonstrated that physical restrain decreases peripheral blood lymphocyte and mitogen-induced proliferation in rats and produces changes in T cell functional capacity [26,10]. In the present study, decreased response of lymphoproliferation was not observed in restrained rats. In addition, no differences were present with variable TAU concentration which could indicate that immune modifications due to physical restrain might be regulated by TAU, possibly through its effect on PKC isoforms.

To better assess the effect of TAUT on the lymphocyte proliferation the TAUT antagonist, β-Ala, was used [27]. In these experiments, at low concentrations of β-Ala lymphoproliferation remains at values similar to basals, but with increasing concentrations, lymphoproliferation significantly increased. It might be that incomplete inhibition of TAUT at low concentrations of β-Ala, in contrast to the effects observed at higher concentrations of this amino acid could be responsible for the differential modulation of lymphocytes proliferation. However, Con A-treated lymphocytes displayed a resistance to the influences of TAU or β-Ala, probably indicating a particular condition of TAUT due to the unspecific activation by the mitogen.

### Plasma levels of interleukine-1β and interleukine-10

**Figure 3:**
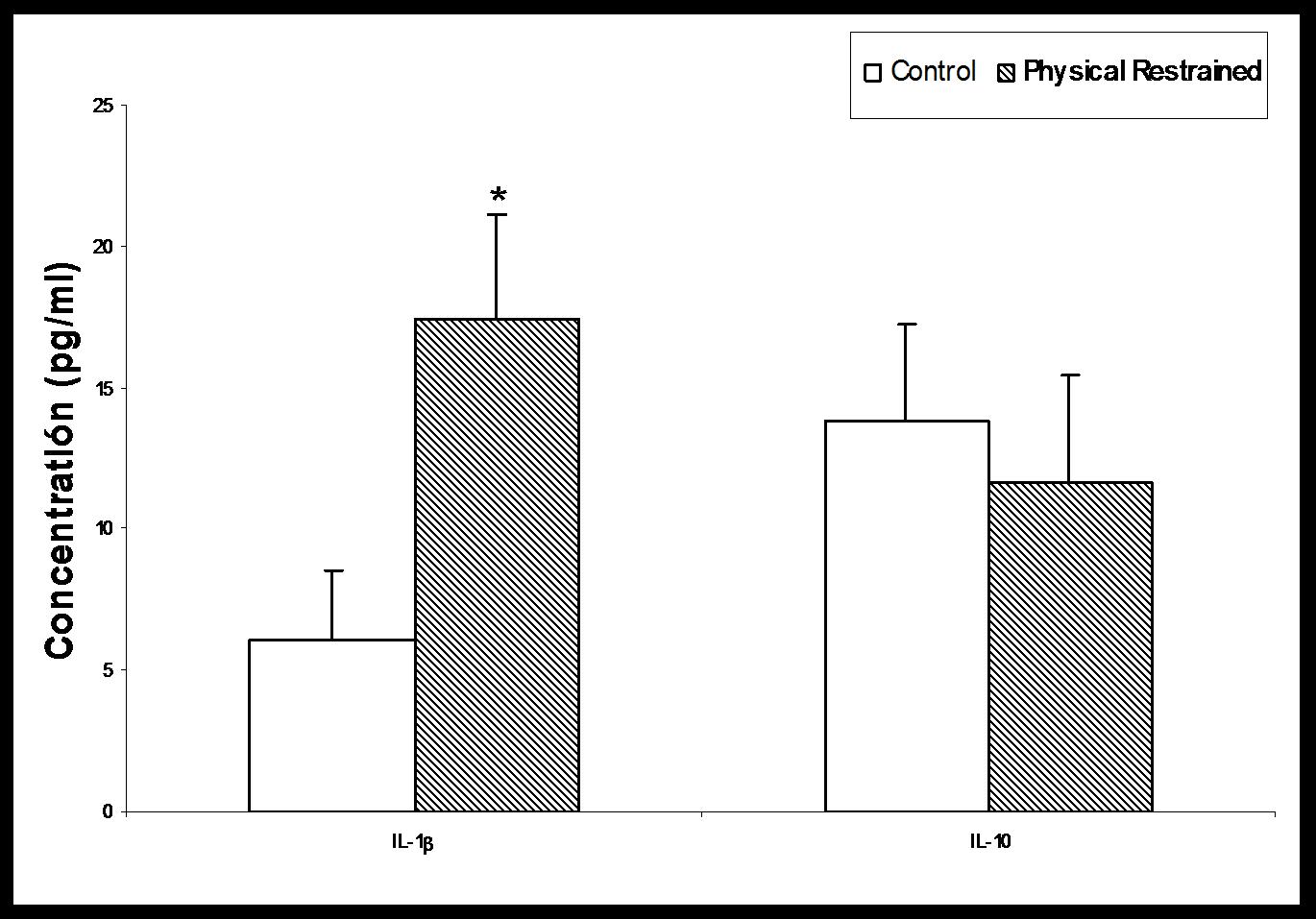
**Plasma concentration of the pro-inflammatory interleukin-1β (IL-1β), and the anti-inflammatory interleukin-10 (IL-10) in Control and in Physical Restrained rats.** Each value represents the mean ± SEM, *n* = 6. P < 0.05 respecting Control.

The concentration of the proinflammatory IL-1β significantly increased in restrained rats, reaching concentrations more than double those of controls, while the anti-inflammatory IL-10 remained unchanged in controls and in restrained rats (Fig. 3).

Physical restraint is known to activate hypothalamic-pituitary-adrenal (HPA) axis, resulting in transiently increased release of glucocorticoids and IL-6 [28,29]. Findings by *in vitro* studies indicate that IL-6 produces upregulation of IL-1β mRNA in human T cells, and this positive feedback by IL-6 on IL-1β secretion occurs after or during stress as a mechanism of partially reducing the downregulatory effect of corticosterone on IL-1β [12]. Moreover, IL-1β, together with TNF-α and IL-6 influence the HPA axis, resulting in increased levels of glucocorticoids in physically restrained rats [30,31]. On the other hand, IL-6 is a pleiotropic interleukin that has multiple functions, including stimulatory effects on proliferation and differentiation of lymphocytes [32], and could be one of the factors influencing lymphoproliferation.

### Plasma and lymphocytes taurine concentration

**Figure 4:**
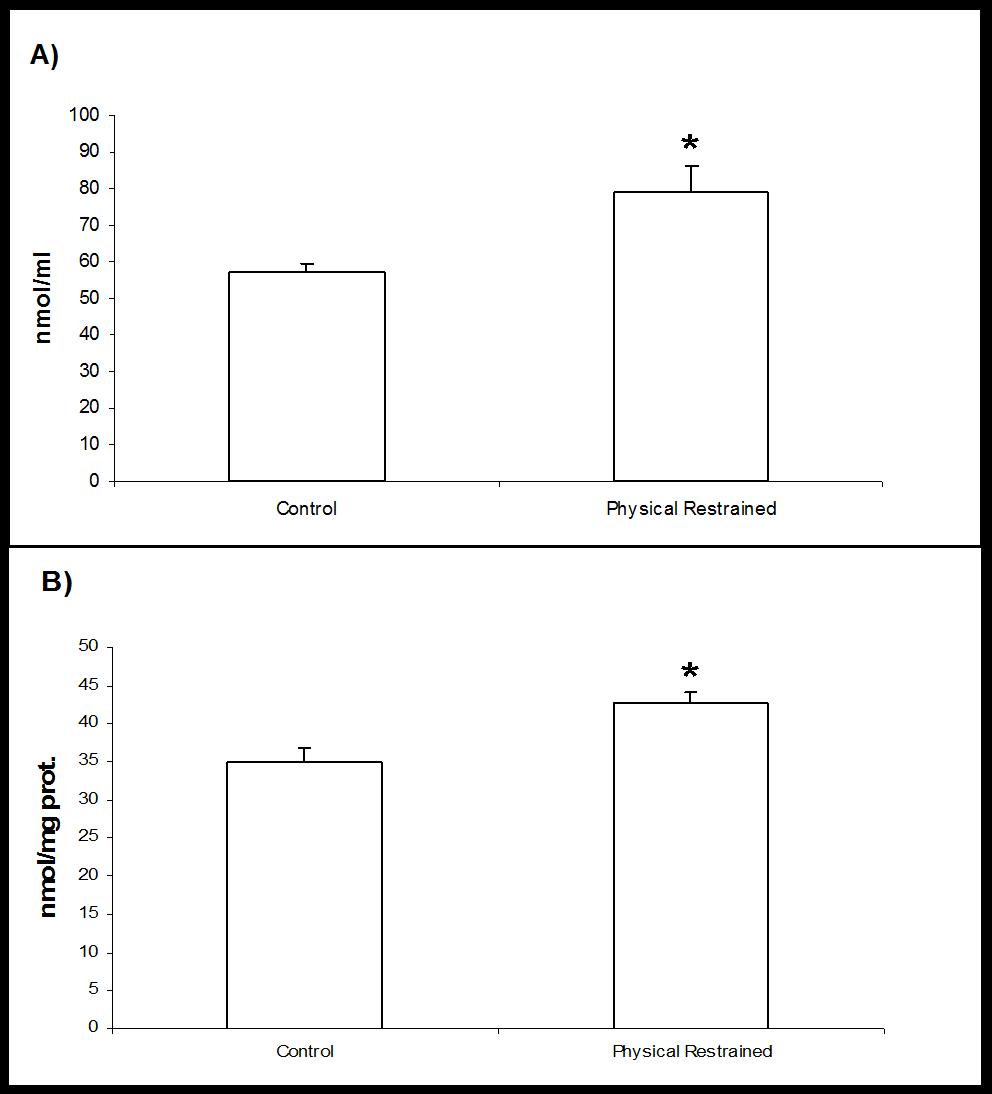
**A) Plasma, and B) lymphocyte concentrations of taurine in Control and in Physical Restrained rats.** Each value represents the mean ± SEM, *n* = 6. P < 0.05 respecting Control.

Figure 4A and 4B represent the levels of TAU in plasma and in lymphocytes, respectively, which significantly increased in restrained rats. Physical restraint leads to hyper-oxidant reaction, and hence in an imbalance between pro-oxidant and anti-oxidant forces [9], as well as increase in proinflamatory cytokines [13]. It is possible that the increase of plasma TAU could be involved in protection against oxidants and in regulation of pro-inflammatory cytokines production by the formation of Tau-Cl.

Previous studies have shown that high concentrations of TAU reduced the expression, activity and nuclear localization of TAUT, whereas low levels increase them [23]. This is due to TAU affects the phosphorylation of specific proteins through PKC [22,33], TAUT, which is regulated by PKC phosphorylation in serine 322 at the post-translational level [34] as an adaptive response to changes in TAU availability. It might be that TAUT could be lower in restrained rats, then produce a temporal increase of TAU in the circulation as a regulatory response. Moreover, the increased values of TAU in lymphocytes could be linked to changes in TAUT.

At the present, studies on TAUT capacity and expression are undertaken in restrained rats.

## Conclusions

Results in culture at different conditions suggest that TAU system plays a differential role in lymphoproliferation, related to concentration, and that physical restraint produces alterations that influence TAU effects. Concentrations of IL-1β were elevated in plasma of rats after physical restraint, which indicate modifications during stress that could trigger pathological changes. The significant increase of TAU levels in plasma and in lymphocytes are probably related to known protective effects of TAU, and might be the result of TAUT changes.

## List of abbreviations

TMB: 3,3´,5,5´-tetramethylbenzidine ; MTT: 3-[4,5-dimetilazol-2-il]-2,5-diphenil-tetrazolio ; β–Alanine (β-Ala); Con A: Concanavalin A; HPLC: High performance liquid chromatography; HPA: Hypothalamic-pituitary-adrenal; IVIC: Instituto Venezolano de Investigaciones Científicas; IL: Interleukin; PBS: Isotonic saline 0.1 M sodium phosphate buffer pH 7.4; PKC: Protein kinase C; RPMI: Roswell Park Memorial Institute Medium 1640; TAU: Taurine; Tau-Cl: Taurine cloramine; TAUT: Taurine transporter; TNF-α: Tumor necrosis factor-α.

## Competing interests

The authors have non-financial competing interests in an exclusive academic way.

## Authors' contributions

FF carried out the experiments, made calculations, participated in the discussion of results, and did most of the writing. FO carried out the HPLC analysis of plasma and lymphocytes TAU levels. LL conceived the study, made the contribution for design, analysis, interpretation of data, discussion, and final writing.
